# Imported Monkeypox from International Traveler, Maryland, USA, 2021

**DOI:** 10.3201/eid2805.220292

**Published:** 2022-05

**Authors:** Varea Costello, Madeleine Sowash, Aahana Gaur, Michael Cardis, Helena Pasieka, Glenn Wortmann, Sheena Ramdeen

**Affiliations:** Walter Reed National Military Medical Center, Bethesda, Maryland, USA (V. Costello);; Uniformed Services University, Bethesda (V. Costello);; Medstar Washington Hospital Center, Washington, DC, USA (M. Sowash, A. Gaur, M. Cardis, H. Pasieka, G. Wortmann, S. Ramdeen)

**Keywords:** monkeypox, international traveler, monkeypox viruses, viruses, travel medicine, global health, orthopoxvirus, zoonoses, Maryland, United States, Nigeria

## Abstract

A case of monkeypox was diagnosed in a returning traveler from Nigeria to Maryland, USA. Prompt infection control measures led to no secondary cases in 40 exposed healthcare workers. Given the global health implications, public health systems should be aware of effective strategies to mitigate the potential spread of monkeypox.

Since the eradication of smallpox, monkeypox has assumed the role of the most prominent orthopoxvirus affecting human communities (*1*). Formerly a rare disease native to Africa, monkeypox is now endemic to countries in western and central Africa, which have faced a resurgence of monkeypox outbreaks over the past decade. More confirmed cases of monkeypox have been diagnosed since 2016 than in the previous 40 years (*2*). Nigeria is in the midst of an ongoing monkeypox outbreak; as of October 2021, a total of 502 cases and 8 deaths from this disease had been reported (*3*). Because of global health implications, in 2017 the World Health Organization and the US Centers for Disease Control and Prevention (CDC) conducted an informal consultation with infectious diseases experts and researchers in countries in Africa to assess the surveillance and outbreak response to monkeypox.

Outside Africa, cases of monkeypox remain rare, but are increasing; 7 international cases have been diagnosed since 2018 (*4*–*6*). In the United States, a case was recently identified in Texas in a traveler returning from Nigeria (*7*). Before that case, the last confirmed monkeypox cases in the United States were in an outbreak involving 47 persons across 6 states; those cases were associated with contact of prairie dogs infected by imported rats from Ghana (*7*). As case rates increase, determining effective public health interventions in preventing secondary spread of monkeypox is critical and a challenge that largely has yet to be confronted in the United States. We describe a case of imported monkeypox in Maryland, USA, and the infection control measures used to prevent additional disease transmission.

## The Study

A 28-year-old man sought care for a diffuse vesicular rash that had developed over the preceding 24–48 hours. He had traveled on a flight from Lagos, Nigeria, and arrived in the United States the day he sought care. While in Nigeria, he visited relatives, stayed in hotel lodging without travel to rural regions, and had no interactions with animals or animal carcasses. During his flight from Lagos, he noticed a burning sensation on his skin, followed by development of discrete vesicles on his forehead and nose, which spread to his arms, trunk, and inner thighs over several hours. He denied having associated symptoms, including fever, chills, or headache.

**Figure 1:**
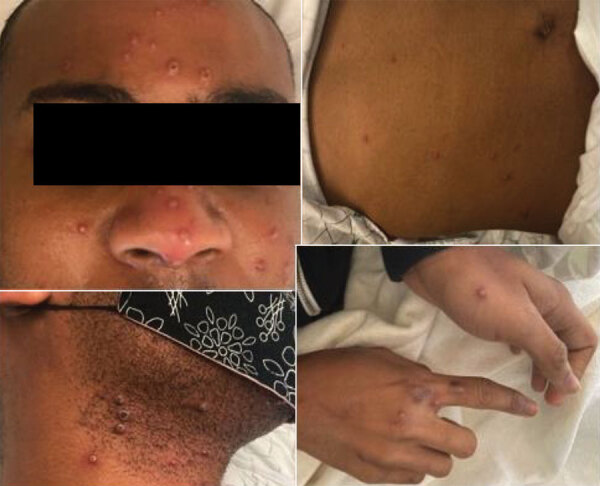
Cutaneous manifestations of imported monkeypox from international traveler, Maryland, USA, 2021. Numerous pustules on erythematous base with some central umbilication and acrofacial propensity are shown.

At examination, we observed right cervical lymphadenopathy and numerous 2–4-mm pustules on an erythematous base. Some of these pustules had central umbilication and were present diffusely with acrofacial propensity, favoring the face, neck, and hands. A few 2–3-mm round erosions were noted on the oral mucosa, and an intact pustule was observed on the lower mucosal lip (Figure 1). The patient was given intravenous acyclovir for empiric treatment of disseminated varicella zoster virus infection, admitted, and subjected to contact and airborne isolation precautions pending further evaluation. Within 24 hours of admission, no new lesions developed, and there was noticeable crusting of several existing vesicles.

**Figure 2:**
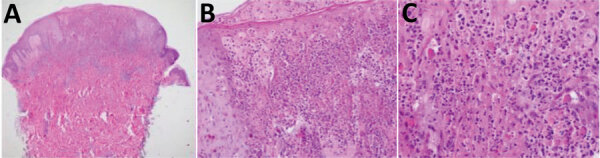
Imported monkeypox from international traveler, Maryland, USA, 2021. A) Epidermal necrosis, reticular necrosis, and vesiculation. In the dermis, a diffuse mixed superficial dermal infiltrate was observed. B, C) Higher magnification views showing dyskeratotic keratinocytes, neutrophil exocytosis, and intracytoplasmic inclusion bodies consistent with Guarnieri bodies in the epidermis. Hematoxylin and eosin stain; original magnification ×4 in panel A, ×20 in panel B, and ×40 in panel C.

We obtained a 4-mm punch biopsy specimen from an intact pustule on the abdomen of the patient. This specimen showed epidermal necrosis, reticular degeneration, and vesiculation by staining with hematoxylin and eosin. We found dyskeratotic keratinocytes, neutrophil exocytosis, and intracytoplasmic inclusion bodies consistent with Guarnieri bodies in the epidermis. We also detected a diffuse, mixed, superficial dermal infiltrate of lymphocytes, histiocytes, neutrophils, and occasional eosinophils (Figure 2).

Based on the travel history of the patient and histopathologic findings, we suspected monkeypox, likely acquired by human contact in the absence of any animal exposures. Additional specimens of the skin lesions were identified by the Maryland Department of Health as non‒variola orthopox by real-time reverse transcription PCR (RT-PCR) and the CDC Laboratory Response Network protocol. CDC used viral culture and RT-PCR to confirm the diagnosis of monkeypox, further identifying the specimen as part of the West African clade, which has driven the outbreak in Nigeria since 2017.

Upon confirmation of the monkeypox diagnosis, we identified all healthcare workers (HCWs) involved in the case and classified 40 as contacts by CDC guidelines (*8*). No HCW met the criteria for high-risk exposure, and no doses of preventive smallpox vaccine were administered. Hospital-based public health officials contacted each HCW daily for 21 days (the duration of incubation period for monkeypox) and instructed them to measure their temperature twice a day and monitor any symptoms. At the conclusion of the surveillance period, we did not detect disease transmission.

## Conclusions

Previously considered a rare zoonotic infection, human monkeypox has reemerged as a clinically serious disease after decades of quiescence. Monkeypox has an overall case-fatality rate of up to 11% (*1*), and increasing human populations have no immunity to poxvirus; therefore, future progress in understanding monkeypox is critical. The World Health Organization Research and Development Blueprint in 2018 classified monkeypox as an emergent disease requiring accelerated research, development, and public health action (*8*). The epidemic potential of monkeypox was demonstrated during the outbreak in the United States in 2003, and had the predominant virus strain been the more virulent and aggressive Congo Basin strain instead of a virus in the West African clade, a higher mortality rate would have been possible (*9*).

Although the public health experience addressing monkeypox in the United States has been limited, this case illustrates the effectiveness of the basic principles of infection control: rapid identification and isolation of the index patient; use of personal protective equipment by HCWs; and thorough contact tracing, including monitoring for secondary cases throughout the totality of the incubation period. Using these interventions alone, our hospital system and community were able to avoid additional disease transmission. Hospital systems should ensure that their healthcare teams, particularly frontline workers, are aware of infection control policies, especially pertaining to patients with possible infectious diseases. In particular, any patient who has a fever and disseminated vesicular or pustular rash should immediately be placed on airborne and contact precautions because these are the typical symptoms associated with orthopoxvirus infection (*10*).

Although vaccination was not required in this case, public health recommendations to prevent secondary disease transmission of monkeypox include the smallpox vaccine (*11*). The vaccine has been estimated to confer 85% protection against monkeypox (*12*), and waning population immunity since routine smallpox vaccine administration ended is postulated to have contributed to its resurgence (*2*). The 2 Food and Drug Administration—approved vaccines are ACAM2000 and JYNNEOS. Either vaccine can be administered preemptively for monkeypox exposures, which is recommended for persons involved in monkeypox outbreak investigations. JYNNEOS is a nonreplicating, live virus, licensed specifically for monkeypox prevention; ACAM2000 is the only recommended vaccine for monkeypox postexposure prophylaxis. On the basis of the effectiveness of postexposure smallpox vaccine, the CDC advises postexposure prophylaxis to high-risk contacts within 4 days and up to 14 days of initial contact with monkeypox (*11*). This intervention has been safely and effectively used by public health officials in the United States, the United Kingdom, and Singapore (*5*,*6*,*13*). 

In addition to smallpox vaccine, vaccinia immune globulin is available and can be used as prophylaxis for severely immunocompromised patients (when smallpox vaccine should be avoided), although the benefit is unclear (*10*). The Food and Drug Administration‒approved antiviral drugs to treat smallpox are tecovirimat and brincidofovir, which can also be used to treat monkeypox, but there are no monkeypox-specific antiviral drugs for treatment or postexposure prophylaxis. Because there are multiple orthopoxvirus vaccine guidance documents, formulation of consolidated recommendations is ongoing (*14*).

In summary, we report a case of monkeypox in a traveler returning to the United States from Nigeria and review infection control measures to prevent secondary cases. Multiple appearances beyond disease-endemic countries indicate that monkeypox has become a relevant travel-related disease, and physicians should remain vigilant in combatting transmission of this virus.
